# Measuring the Implementation of Behavioral Intervention Technologies: Recharacterization of Established Outcomes

**DOI:** 10.2196/11752

**Published:** 2019-01-25

**Authors:** Eric DA Hermes, Aaron R Lyon, Stephen M Schueller, Joseph E Glass

**Affiliations:** 1 Veterans Affairs Connecticut Healthcare System West Haven, CT United States; 2 Department of Psychiatry Yale University School of Medicine New Haven, CT United States; 3 Department of Psychiatry and Behavioral Sciences University of Washington School of Medicine Seattle, WA United States; 4 Department of Psychological Science University of California at Irvine Irvine, CA United States; 5 Kaiser Permanente Washington Health Research Institute Seattle, WA United States

**Keywords:** mobile applications, behavior therapy, technology, internet, telemedicine, diffusion of innovation, translational medical research, outcome assessment (health care), review, implementation, behavioral intervention technology

## Abstract

Behavioral intervention technologies (BITs) are websites, software, mobile apps, and sensors designed to help users address or change behaviors, cognitions, and emotional states. BITs have the potential to transform health care delivery, and early research has produced promising findings of efficacy. BITs also favor new models of health care delivery and provide novel data sources for measurement. However, there are few examples of successful BIT implementation and a lack of consensus on as well as inadequate descriptions of BIT implementation measurement. The aim of this viewpoint paper is to provide an overview and characterization of implementation outcomes for the study of BIT use in routine practice settings. Eight outcomes for the evaluation of implementation have been previously described: acceptability, adoption, appropriateness, feasibility, fidelity, implementation cost, penetration, and sustainability. In a proposed recharacterization of these outcomes with respect to BIT implementation, definitions are clarified, expansions to the level of analysis are identified, and unique measurement characteristics are discussed. Differences between BIT development and implementation, an increased focus on consumer-level outcomes, the expansion of providers who support BIT use, and the blending of BITs with traditional health care services are specifically discussed. BITs have the potential to transform health care delivery. Realizing this potential, however, will hinge on high-quality research that consistently and accurately measures how well such technologies have been integrated into health services. This overview and characterization of implementation outcomes support BIT research by identifying and proposing solutions for key theoretical and practical measurement challenges.

## Introduction

### Behavioral Intervention Technology

A broad range of health information technologies are increasingly used in the delivery of health care to expand access, increase the effectiveness of care, and improve the productivity of health systems [1,2]. This article focuses on a subset of health information technology developed to intervene in a wide range of behavioral, psychosocial, or chronic health conditions, termed *behavioral health* conditions, by assisting the user to change behaviors, cognitions, and emotional states [3]. The term behavioral intervention technology (BIT) is used to refer to these interventions, although alternative terms such as eHealth, mobile health, *digital treatments*, and *internet interventions* are also used [4].

BITs are interventions delivered over computer software, internet websites, mobile apps, and wearable devices [2]. Such programs present material in varied formats, including audio, video, text, or games. BITs may include symptom assessments, didactic lessons, passive sensing, and feedback systems that record and present a range of user activities and responses. BITs are used primarily by health care consumers but are also accessed by providers and others involved in the delivery of care. There are efficacious BITs for the treatment of most common behavioral health conditions, including depression, insomnia, substance use disorders, diabetes, and hypertension (Table 1) [5].

BITs represent an example of the move toward a more patient-centered health care system by empowering consumers to participate in their own care [1]. Some BITs provide novel data streams that providers can use to monitor patient outcomes, inform decision making, or improve care coordination. BITs may also increase access to and the convenience of care by reducing longstanding barriers such as travel, scheduling, and stigma while also potentially reducing health care costs [16-18]. Consequently, the capacity of BITs to support the so-called *triple aim* of health care reform (improved patient experience, population health, and costs) has spurred rapid growth in their development [19].

Nearly two decades of research on BITs has produced promising initial findings of efficacy; however, there are few examples of successful implementation and sustainment in routine practice settings [4,20]. For example, in a recent large pragmatic effectiveness trial in the United Kingdom, consumers used BITs for depression much less frequently than recommended, and the trial failed to demonstrate improvement in outcomes. However, a subsequent trial adding telephone support to BIT use did improve outcomes [21,22]. Given that use of such programs outside of research settings has been limited, there is a growing recognition that BITs will not revolutionize health care, without a better understanding of the factors associated with their implementation.

### Objectives

In what has become a leading reference for the measurement of health service implementation, Proctor et al defined 8 outcomes for the measurement of implementation: acceptability, adoption, appropriateness, feasibility, fidelity, implementation cost, penetration, and sustainability (or sustainment) [23]. The authors called for further work to conceptualize implementation outcomes across contexts and interventions. We heed this call and argue that traditional implementation outcomes must be recharacterized in light of the unique aspects of BITs and the models of health care delivery they favor. The objective of this article is to advance and clarify the diverse outcomes used to study the implementation of BITs.

This paper both relies on previous work in the area of BIT implementation and fills gaps in that work. Prior work has discussed the use of frameworks and theories to develop and implement health information technology [24,25]. This paper provides needed guidance on measuring the implementation of such technology. Moreover, several systematic reviews have evaluated how implementation outcomes have been measured for a subset of programs aimed at specific populations or a subset of implementation outcomes such as adoption and fidelity [4,26-28]. This paper provides a discussion of the full range of implementation outcomes with respect to a full range of BITs, addressing a host of behavioral health concerns. The goal of this article is to advance the evaluation of BIT implementation, with the hope of improving future implementation efforts and identifying key factors for successful implementation. We begin with a discussion of the types of services BITs provide to frame the argument. We then discuss each of Proctor et al’s outcomes with regard to BIT implementation, followed by a discussion of measurement recommendations and future directions for BIT implementation work.

### The Continuum of Health Services Supported by Behavioral Intervention Technology

**Figure 1:**
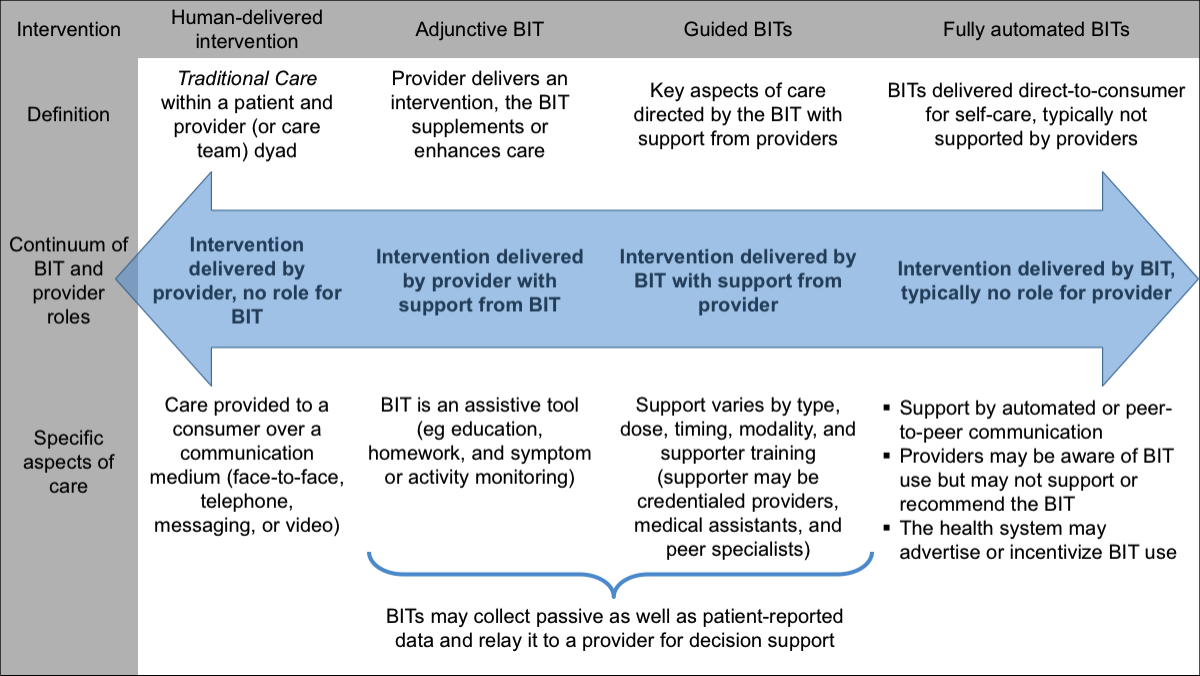
Continuum of support for delivering behavioral intervention technologies (BITs).

The amount of clinical support provided to consumers using BITs has critical implications for the measurement of implementation outcomes. Some BITs are primarily consumer-facing products, designed to be used by consumers alone for care that is not directed by a health care provider (*self-care* or *fully automated* BITs, eg, MoodGYM [8,29]). Other BITs are intended to be used as a component of care that is delivered by a provider (*adjunctive* BITs; eg, the CBTi Coach mobile app [developed by US department of Veteran Affairs, Veterans Health Administration] as an adjunct to provider-led cognitive behavioral therapy for insomnia) [30]. As implementation research is focused on how practices are integrated into care settings, the level of provider integration with BIT use has critical implications for implementation outcome measurement. A description of the types of BITs and how they vary according to different levels of provider involvement is given in Figure 1, adapted from work by Muñoz (2017) [31].

**Table 1:** Examples of behavioral intervention technology programs.

Behavioral intervention technologies program	Program objective	Platform	Evidence
BlueStar or WellDoc	Diabetes management	Mobile	[6]
MoodGym	Depression	Web	[7,8]
Sleep Healthy Using the Internet (SHUTi)	Insomnia	Web	[9-11]
PTSD Coach and PE Coach	Posttraumatic stress disorder symptom tracking and treatment support	Mobile	[12-14]
reSET or Therapeutic Educational System	Substance use disorders	Mobile	[15]

## Recharacterization of Implementation Outcomes for Behavioral Intervention Technology Implementation

### Overview of Recharacterization

In a major advancement for implementation research, Proctor et al characterized 8 separate outcomes for the measurement of implementation [23]. In the following sections and Table 2, these implementation outcomes are recharacterized with respect to the unique aspects of BITs: the data streams produced by BITs, the continuum of support with which BITs are often employed (Figure 1), and the levels at which BIT implementation outcomes are analyzed (consumer, provider, administrator, and organization).

There are 3 important points to contextualize this recharacterization of BIT implementation outcomes. First, unless otherwise noted, we conceptualize implementation outcomes as applying to the clinical *intervention* —the BIT and the clinical activities intended to guide BIT use—rather than on the strategy or process used to implement the BIT. For example, the outcome of fidelity is limited to intervention fidelity (ie, the extent to which the BIT is delivered as intended) and not implementation fidelity (ie, the extent to which the strategy or process used to implement the BIT is followed as intended). Second, we consider BITs as interventions themselves rather than implementation strategies for other evidence-based practices (eg, an evidence-based self-care BIT for insomnia is an intervention and not an implementation strategy for cognitive behavioral therapy for insomnia). Although technology may intersect with the strategy used to implement an evidence-based practice, we do not consider the act of digitizing treatment to be an implementation strategy, as BITs require implementation strategies themselves to ensure successful implementation. Third, the construct of usability is pervasive in the technology design literature and partially overlaps with the implementation outcomes discussed. Building on the International Organization for Standardization’s definition, we define BIT usability as the extent to which a BIT can be used to achieve the program’s goals with accuracy, completeness, efficiency, and satisfaction in a specified context [32]. The concept of usability can be applied and assessed in all phases of BIT testing, from intervention development to preimplementation, implementation, and sustainment. BIT usability and its relationship with implementation outcomes is discussed more extensively below.

### Implementation Outcomes

#### Acceptability

Acceptability is the extent to which an innovation is agreeable, palatable, or satisfactory to a stakeholder [23]. For BITs, acceptability can be evaluated among a variety of stakeholders (consumers, providers, administrators, and policy makers) and for a variety of BIT aspects, from intervention content to program appearance. A recent 2018 systematic review of implementation measurement for technology-based mental health interventions found that acceptability was the outcome most frequently measured, commonly via project-specific, nonvalidated measures [28].

**Table 2:** Characterization of behavioral intervention technology implementation outcomes.

Outcome and definition [23]		Level of analysis in BIT^a^ studies	Measurement objective and process	Example of BIT outcome measurement
**Acceptability**				
	Perception among stakeholders that a given evidence-based practice is useful or satisfactory	Individual provider, consumer, or administrator	Objective: assessment of the extent to which BIT aligns with expectations of an agreeable user experience Process: survey, interview, focus group, and direct observation usability testing	Mares et al (2016): qualitative methods used to assess initial consumer and provider expectations [33] Milward et al (2017): focus groups assessed the extent to which features were acceptable in terms of content, features, and design [34]
**Adoption**				
	Intention, decision, or initiation to use an evidence-based practice	Individual provider, consumer, or administrator	Objective: assessment of *actual system use* or *behavioral intention to use* Process: passive data collection of BIT use [35]	Gilbody et al (2016): to measure consumer-level adoption, log-in records were used to identify number of participants who accessed programs [22]
**Appropriateness**				
	Perceived fit, relevance, or compatibility of the evidence-based practice to a given context	Individual provider, consumer, or administrator Organization	Objective: assessment of perceived BIT fit with the context Process: survey, interview, focus group, direct observation usability testing, and workflow studies	Lyon et al (2016): evaluated school-based practitioner workflows and current technology use practices to determine the appropriateness of a digital measurement feedback system and identified areas for BIT redesign [36]
**Feasibility**				
	Extent to which an evidence-based practice can be successfully used or conducted within a given context	Individual provider, consumer, or administrator Organization	Objective: in vivo assessment of the extent to which a BIT can be used by consumers or providers in a specific setting Process: Passive data collection of BIT use, survey, and structured observation studies	Kumar et al (2018): using program use data collected via BIT, the feasibility of implementing a mobile app for consumers and a provider-facing dashboard was tested in 4 outpatient clinics [37]
**Fidelity**				
	Extent to which implementation results in an evidence-based practice being delivered as intended	Individual consumer or provider Organization	Objective: measuring adherence, dose, or quality of BIT use with respect to the developer’s intentions for use Process: passive data collection of BIT use	Calear et al (2013): reported high adherence associated with improved clinical outcomes in a hybrid implementation effectiveness study [7] Sineath et al (2017): developed and tested a fidelity protocol for a diet and lifestyle monitoring BIT that involved coaching [38]
**Implementation cost**				
	Costs associated with implementing an evidence-based practice	Organization	Objective: intervention development costs, maintenance and versioning costs, implementation strategy costs, and operational costs Process: cost analysis, interviews, and budgetary or administrative databases	Quanbeck al (2018): measured implementation strategy costs for implementation coaching time and site visits needed to help 3 organizations integrate BIT into practice [39]
				
				
				
				
**Penetration**				
	The integration of an evidence-based practice within a service setting (organization) and its subsystems	Organization	Objective: measuring the number of consumers or providers using BITs among those eligible or trained to engage in BIT Process: passive data collection of BIT use and electronic health record data	Titov et al (2015): measured the proportion of individuals in a defined consumer population completing lessons in 4 different BITs [40]
**Sustainability**				
	The extent to which a newly implemented evidence-based practice is maintained or institutionalized within a service setting’s ongoing, stable operations	Administrators Organization	Objectives: measuring ongoing BIT use, change in funding streams, saturation within the organization, and inclusion in routine reports Process: passive data collection of BIT use, administrative or budgetary databases, oversight committee reports, and policy and training documents	Carlfjord et al (2013): measured continued BIT delivery after active implementation [32] Quanbeck et al (2018): measured whether or not the health system continued to offer BIT after research funding for an implementation trial ended [39]

^a^BIT: behavioral intervention technology.

The most well-known model of health information technology acceptability is the Technology Acceptance Model (TAM), in which the perceived usefulness and ease of use of the technology is assessed, along with a number of associated constructs such as prior experience, output quality, and social influence [35]. The concept of technology usability clearly overlaps with acceptability and is included as a major construct in the TAM and similar models. However, usability is a broader concept, encompassing additional issues such as likelihood of error and efficiency of use. In BIT research, usability is most often measured by self-report, which may be the component of usability that shares the most conceptual overlap with acceptability. Self-report measures of usability, such as the System Usability Scale, best track the notion of *perceived* usability (eg, Does a user believe that a BIT will be able to help them achieve a goal?) rather than *actual* usability, which involves observing whether those goals are achieved [41]. Validated measures such as the Attitudes Towards Psychological Online Interventions questionnaire also include assessments of acceptability [42]. Schroder et al (2017) used this measure to assess the acceptability of a BIT for depression among both consumers and providers in a randomized controlled trial [43].

#### Adoption

Proctor et al define adoption as the intention, decision, or initiation of use for an evidence-based practice, characterizing it at the level of the provider or organization [23]. Given the self-care nature of guided and fully automated BITs, this level of analysis is expanded to that of the consumer. The concept of adoption aligns with constructs of *actual system use* or b*ehavioral intention to use* in models such as TAM, for both consumers and providers [35].

The measurement of BIT adoption primarily relies on data gathered by the program, which is either passively collected (eg, log-in timestamp) or input by the consumer (eg, self-report symptom measure). However, direct observation and surveys of BIT use can occur. The amount and relative ease of data extraction from BITs can make intentions, decisions, or initiations more evident and accurate, without the need for labor-intensive data collection processes. In a large-scale naturalistic trial, Gilbody et al used log-in records to identify the number of study participants who accessed BITs for depression [22].

#### Appropriateness

Appropriateness is the perceived fit or compatibility of an innovation with a practice setting or context [23]. The key concept is the *perception* of fit, making the measurement of appropriateness more relevant to initial phases of implementation, but not strictly limited to these phases. Fit can be assessed at the organizational level (eg, alignment with workflow and policies) or the individual level (eg, alignment with providers’ or consumers’ attitudes, needs, and background). When evaluating implementation outcomes for BITs, we add administrators, given that BIT components such as dashboards can be accessed by administrators.

It is important to clearly specify which individuals or groups are intended to use a BIT and to elicit their needs when determining appropriateness. Usability evaluations can be employed to capture expectations, cognitions, and emotional responses of potential users [44]. Formative usability assessment seeks to assess the perceived contextual fit of new products with their destination context before actual implementation. Laboratory-based usability testing is particularly relevant to the initial assessment of appropriateness. Assessing the existing health information technology infrastructure, workflow, and use, as well as the potential interoperability of the BIT at a site, are key factors later in the process [45,46]. For instance, Lyon et al (2016) evaluated school-based practitioner workflows and current technology use practices to determine the appropriateness of a digital measurement feedback system before its implementation [36].

#### Feasibility

Feasibility is the extent to which a new evidence-based practice can be successfully used or conducted in a setting. Feasibility differs from appropriateness as it is typically based on direct observation of stakeholder’s experiences and practical concerns derived during or after implementation. Traditional conceptualizations of feasibility assessment include data collected from service providers and organizations and are rooted in the assumption that new innovations pass directly through providers to consumers [23]. For BITs, feasibility must be expanded to accommodate scenarios such as those involving fully automated BITs that may reach consumers with limited mediation from providers or the health system.

Standardized measures of innovation feasibility remain applicable to BITs [47]. In addition, although assessment of the acceptability and appropriateness of BITs are associated with formative usability testing, the assessment of BIT feasibility can leverage summative testing to evaluate how well full products meet their specified objectives in the actual context of use [48,49]. Such assessment might include evaluation of user awareness of key features of a BIT, actual compatibility with provider workflows, or user workarounds to account for unaddressed design issues. As feasibility is associated with users’ actual experiences with an innovation in a specific context, in vivo usability testing is particularly relevant. Passive data collection opportunities surrounding feasibility may include frequency, time of day, or other contexts in which key BIT features are used. For instance, Lappalainen et al evaluated the feasibility of an adjunctive BIT for workplace stress reduction by determining how frequently users accessed an Web-based portal during the study [50].

#### Fidelity

Fidelity is the extent to which an intervention is used as intended and measured across several domains: protocol adherence, dose or amount delivered, and quality of delivery [23]. Protocol adherence to a BIT can be conceptualized as the functions of the program that were used, dose as the frequency of program use, and quality as whether the BIT was delivered correctly or for the intended purpose. The standards for these measurements should be based on the intentions of the BIT developers or empirical findings from efficacy testing. Therefore, fidelity to BITs suggests a match between the intended use of the program, termed *expected use* or *clinically meaningful use*, and its actual use by consumers [51]. Fidelity is traditionally measured at the provider level. However, the consumer level may be favored for BITs, especially for fully automated technologies.

BITs offer the opportunity to collect an extensive amount of passive data, which can be used to measure fidelity. Frequency metrics such as the number of log-ins, clicks, or task completions, as well as viewing times may best serve as measures of dose, whereas data representing the breadth of specific program functions used (eg, viewing of videos, completion of measures, and use of Web-based diaries) can serve as measures of adherence. Donkin et al provide a systematic review that includes measures of dose and adherence [27]. As for quality, BITs are a technology-driven platform, and they typically present information, direct activities, and make assessments in a uniform manner, which may provide better quality consistency compared with traditional human delivered care. Examples of measuring the quality dimension of fidelity include determining whether the BIT was used in a group of consumers with the appropriate condition, if the content of diary or self-report entries comply with the information requested, or whether passive sensors accurately assessed what was intended.

The measurement of certain aspects of fidelity, such as protocol adherence, may be less relevant for programs where consumers have more choice in when and how much they engage with the intervention. Determining *expected use* or *clinically meaningful use* with respect to BIT implementation is especially relevant and may be especially difficult for BITs developed as programs where users can openly navigate to browse information, activities, and assessments at their own discretion. This format differs from sequential navigation programs in which users are directed through a structured format to a specified endpoint [52]. Open navigation designs are increasingly common because of consumer desires for flexibility. This issue of clinically meaningful use and the level of fidelity associated with successful implementation is also relevant for programs used as an adjunct to provider-led care, where clinical outcomes may be associated more with provider activities than use of the BIT. A prime example is the Veterans Health Administration program *PTSD Coach*, a mobile phone BIT used in conjunction with provider-facilitated therapies for the treatment of posttraumatic stress disorder. The program has multiple functions, including the capability to monitor symptoms, learn relaxation techniques, and track appointments [12,13]. Lack of clarity around what constitutes *expected use* or *clinically meaningful use* might be one reason that research linking platform use and clinical outcomes has produced mixed findings [26,27].

#### Implementation Cost

Proctor et al identify 3 factors that drive costs in implementation research: the intervention, the setting of service delivery, and the strategy used to implement the intervention [23]. Intervention development and implementation may be blurred with respect to BITs, making these distinctions somewhat artificial. The decision to account for specific implementation costs in operational or implementation budgets will likely vary by organization but should be made clear in cost analyses.

BIT intervention costs include those associated with program development, testing, versioning, and maintenance. Program development and refinement also occurs with traditional face-to-face psychosocial interventions, but versioning and maintenance may be more applicable to BITs (eg, version 2.0 of a program) [53]. When developed outside the health system, intervention costs include the price paid by the health system for the BIT or to develop initial and subsequent versions of the BIT.

Costs associated with the context of service delivery will vary according to the implementation site’s size, complexity, overhead, and how much adaptation of the site’s current health information technology platform is needed. For instance, Quanbeck et al measured system operating costs, which they saw as distinct from implementation costs, including staff time for introducing the BIT to patients and monitoring data produced by the BIT, technical support costs, and ongoing costs such as server hosting [39].

Implementation strategy costs are those incurred directly from the strategies used to employ the BIT, and vary depending on where the BIT lies on the continuum of support defined in Figure 1 [54]. Guided BITs may require more time to educate providers and consumers, facilitate engagement, and support use. In addition, resources such as advertising materials, educational materials, registries, clinical reminders, and decision support tools may be needed. Implementation strategies for fully automated BITs likely incur fewer costs given that providers do not require training and support to facilitate BIT use. In such cases, strategy costs may focus primarily on advertisement and other materials facilitating engagement.

#### Penetration

Penetration is the integration of a practice within a service setting and is measured within an organization. Some BITs allow for an expansion of service providers to include an array of providers who may support consumers during BIT use [23]. Other BITs may follow fully automated formats circumventing the involvement of providers and allowing for implementation outside of traditional health care systems. Thus, BITs may expand the breadth of providers for which penetration is measured and expand the reach of health care organizations beyond their *brick and mortar* facilities to draw in new consumer groups.

Penetration is primarily a summative implementation outcome traditionally measured as the number of providers who deliver a service among the total number of providers trained in or expected to deliver the service and/or the number of consumers engaging in a service among the number eligible to engage in a service within an organization [23]. Such traditional measurement applies well to adjunctive BITs. However, for guided BITs, penetration at the service provider level should be reconceptualized to include personnel specially trained to support BIT use, which comprises traditional health care delivery professionals as well as health technicians, administrative personnel, and peers [29]. The decision to include such adjunct providers in the larger denominator of providers, or measure penetration separately according to provider role is up to the researcher. We note that in such cases, the number of adjunct providers trained to support BIT use may be small, especially if they are centralized to provide remote support across a health care organization. Consumer-level penetration or fidelity to BIT delivery may be a more impactful measure in such cases.

Some fully automated BITs may be implemented outside the traditional health system contexts at the consumer level, either *adjacent* to health care systems to specifically targeted populations or disseminated worldwide as in Massive Open Online Interventions (MOOIs), where specific targeting strategies are not used [55]. In the case of targeted populations, the denominator for penetration must be selected from those for which the intervention is clinically appropriate and to which the outreach efforts for the intervention were aimed. Measuring MOOI penetration may be difficult, as the denominator cannot be precisely calculated, and estimated penetration, based on the proportion reached given the prevalence of a targeted condition, may be more appropriate.

#### Sustainability

Sustainability is the degree to which an implemented treatment is maintained, institutionalized, or integrated within a service setting. For BITs, sustainment is characterized at the level of the organization or setting. Proctor et al distinguished 3 constructs for the measurement of sustainability: passage, niche saturation, and inclusion in cycles or routines [23].

The implementation of BITs in US health care systems has primarily taken place as effectiveness trials and pilot programs. Thus, sustainment is the least well-documented BIT implementation outcome [4]. As BIT use matures, passage from research and development funding streams to permanent organizational funding will be important to measure. In addition, the integration of BITs with pre-existing organizational health information technology such as electronic health records or other patient or provider dashboards will be an important measure of niche saturation. Similarly, the addition of metrics on BIT use and clinical outcomes in routine administrative reports will be an important measure of inclusion in cycles or routines.

With respect to fully automated BITs implemented outside of traditional health care settings, the analysis of sustainment beyond simple continued adoption may be difficult because of the novel organizational cultures in these contexts. As a hypothetical example, analysis of the sustainment of a BIT aimed at reducing alcohol intake among veterans and implemented through social media by a veteran service organization may rely on analysis of the organization’s continued budget for advertising and program maintenance as a gauge of sustainment, in addition to routine adoption or fidelity measures.

An additional unique aspect of BITs is the concept of intervention versioning, whereby there is a continuous evolution of an intervention through frequent program updates [56]. Some changes are small (ie, bug fixes or cosmetic improvements), whereas others may include substantial changes to program content and function. Measurement of implementation and specifically sustainment of BIT use must account for such changes. At a minimum, the timing and content of changes should be tracked and associated with any changes in BIT use and clinical outcome.

## Recommendations for Behavioral Intervention Technology Implementation Measurement

A key factor in BIT implementation measurement involves the level of provider integration. Fully automated BITs are platforms for patient-centered self-care, the implementation of which may circumvent traditional health care organizations. As such, implementation measurement for fully automated BITs should emphasize the consumer level for outcomes such as adoption, and the organizational level, which may include nontraditional health care delivery organizations, for outcomes such as cost and sustainment. In guided BITs, care is directed by the BIT and support is delivered by a provider. Implementation outcomes are still primarily measured at the consumer level. However, the measurement of provider or coach activities is also important, especially with respect to fidelity, implementation cost, and sustainment. In adjunctive BITs, BIT use constitutes an evidence-based practice that supports aspects of provider-led care. The traditional provider-level focus of implementation outcome measurement still applies. However, the BIT may allow unique ways to measure such outcomes. For example, the use of an adjunctive BIT might increase fidelity to an evidence-based practice as well as provide objective information on that fidelity through passive data collection. On the basis of the above discussion of BIT implementation outcomes, we have identified additional recommendations for the measurement of BIT implementation in Textbox 1 and in the proposed agenda for BIT implementation research below.

**Implementation outcome and recommendations for measuring behavioral intervention technology (BIT) implementation.**

Implementation outcome and recommendations

Acceptability

- Articulate measurement distinctions between acceptability and usability.
- Use validated measures of acceptability.

Adoption

- Expand measurement to the consumer level, in addition to providers in the case of guided and adjunctive BITs.
- Define and measure adoption using BIT data streams before and during the implementation effort.

Appropriateness

- Evaluate usability or workflow to assess appropriateness.
- Assess user perceptions of appropriateness in the initial phases of implementation.

Feasibility

- Measure using data acquired through BIT data streams at the consumer and provider levels.
- Define and measure feasibility before and during the implementation effort.

Fidelity

- Consider measurement approaches that include dose, adherence, or quality.
- Measure at the consumer level to ensure the course of treatment (BIT usage) is followed as intended.
- Measure at the provider level to ensure the adequacy of BIT guidance, support, or coaching.
- Base standards for fidelity on intentions of developers or empirical findings from BIT testing.
- Develop clear fidelity standards for openly navigated programs.

Implementation cost

- Assess BIT costs (development, testing, and versioning); context costs; and implementation strategy costs.

Penetration

- Measure primarily among consumers and providers.
- Include providers who support BIT use in guided BITs.
- Include groups targeted for BIT use in the denominator.

Sustainability

- Measure passage to permanent funding, integration with pre-existing technology, and inclusion in routine reports, in addition to continued BIT use beyond active implementation.
- Attend explicitly to BIT versioning and updates.

## Agenda for Behavioral Intervention Technology Implementation Research

### Agenda Overview

BITs are novel platforms for behavioral health interventions that promote patient-centered care by blending technology- supported self-care with traditional health services led by providers. In spite of the potential benefits of BITs and the well-documented efficacy of many BITs, their successful implementation has faced considerable challenges [22,28]. To help advance the study of factors associated with successful BIT implementation, there should first be consensus on the measurement of BIT implementation outcomes. To this end, we have selected several characteristics of BIT implementation on which future research should be based.

### Focus on Implementation Outcomes at the Consumer Level

BITs are 1 aspect of the current transition in health care delivery from services focused on traditional provider-centered face-to-face care in *brick-and-mortar* facilities to patient- centered services that include self-care [1,57]. This new form of care may be better described as *brick-and-click*, as it incorporates the traditional provider-led care in health care facilities, with the *clicks* associated with consumer-led health information technology use. Consequently, the implementation of patient-centered digital interventions will require greater focus on and more precise measurement of consumer-level implementation outcomes while continuing to assess the provider- and organizational-level implementation outcomes that have been the traditional focus.

For example, there is a need for more precise characterization of the different aspects of BIT adoption and fidelity. BIT developers and implementation researchers must clearly define what consumer-level adoption means for a specific BIT or type of BIT. Is the most appropriate measure of consumer adoption an initial program log-in, a specific number of clicks or pages visited, or a threshold of elapsed engagement time? Alternatively, stages, milestones, or levels of adoption can be quantified based on the engagement in or completion of certain activities, similar to the stages of implementation completion [58].

Fidelity must also be clearly defined for a given BIT, especially for programs that allow users to access a range of resources in a self-directed manner. The level of use that constitutes adequate fidelity should ideally be specified before program implementation. For example, fidelity to a BIT modeled after a manualized cognitive behavioral therapy for insomnia may be relatively straightforward: completing the program’s 6 sessions, associated homework, and daily sleep diary entries [11]. However, determining adequate fidelity to a BIT that does not dictate how or how much it should be used, such as the *Vets Prevail* program where consumers access a range of activities for depression and anxiety symptoms in a nonlinear fashion, may be more difficult [59]. Ultimately, the association between fidelity and clinical outcomes must be measured [27].

### Behavioral Intervention Technology Usability Assessment and Implementation Outcomes

There is a conceptual and functional overlap between BIT usability assessment and implementation outcomes. We see usability as a broad concept centered primarily in program design and development phases but with clear elements overlapping with the early stages of implementation. We hypothesize predictive associations between (1) usability assessment in the development of BITs; (2) implementation outcomes of acceptability, appropriateness, and feasibility; and (3) subsequent downstream implementation outcomes of BIT adoption, penetration, and sustainment [60]. These hypotheses need empirical investigation. Moreover, the overlap between the conceptual and practical aspects of usability and implementation outcomes highlights potential for ambiguity among the implementation outcomes described. As others have proposed, there may be theoretical divisions between these outcomes but little empirical evidence or practical means to measure distinctions between them [61]. For instance, some have determined feasibility by measuring the frequency of logging in to a BIT portal, whereas others may consider this a measure of consumer adoption [50].

### Rapid Behavioral Intervention Technology Development

Similarly, the transition from intervention development and testing to implementation is accelerated and more iterative for BITs compared with traditional services [56]. In some ways, the technology aspects of BITs, including program updates and versioning may allow for more consistent delivery of services, but rapid development and versioning poses problems for evaluation and implementation. For example, the relative ease of deployment may have led to the attempted implementation of some BIT programs or updates without evidence of usability or efficacy [62,63]. Such situations may lead to innovation fatigue among consumers and providers, potentially undermining successful sustainment [64]. The timing of implementation with respect to evidence of innovation usability and efficacy is a topic of interest in implementation science as a whole and deserves more investigation with respect to BITs.

### Expansion of Providers Who Support Behavioral Intervention Technology use

The implementation of BITs will also broaden the definition of care providers to include individuals who support care through BITs, such as health technicians, administrative personnel, peers, and other paraprofessionals [65,29]. Implementation outcome measurement, especially fidelity, must accommodate the activity and perspectives of these individuals. The measurement of this activity may be nuanced or difficult, as BITs may be implemented outside of traditional health care contexts, the consumer and provider may be geographically separated, the provider may not be employed by the health system, or communication maybe infrequent or through nontraditional means such as short message service text messages. Implementation researchers must be aware of these contingencies and clearly define the roles of different providers as well as metrics to capture their actions.

### Combining Behavioral Intervention Technologies With Traditional Health Care Services

As described in Figure 1, BITs integrate their treatment function across a continuum of human involvement, from provider-led services (adjunctive BITs) to consumer-facing self-care services (fully automated BITs). Future research should better characterize the breadth of this continuum as well as ramifications for BIT implementation outcome measurement, implementation strategy development, and the empirical determination of clinically meaningful use. For example, implementing adjunctive BITs requires acceptance and adoption of technology by the consumer, provider, and organization, with relatively little change in individual roles. The implementation of such programs may not be unlike the implementation of similar adjunctive services such as blood pressure or glucose monitoring devices. However, BITs that support a high degree of self-care, such as guided BITs, will likely require a greater degree of change to the basic function and workflow of health care delivery organizations, requiring more robust implementation strategies. Such relationships should be investigated in future BIT implementation research.

Stepped care models, where low-intensity treatment is initiated first followed by elevations in treatment intensity as needed, are an intuitive approach to integrating BITs in health care systems, where BITs serve as the initial stage before more intensive face-to-face treatment [66]. It is also possible to integrate stepped care across the continuum of BIT support. For instance, fully automated BITs could serve as a low-intensity initial step, with patients being stepped up to more intensive coaching or other forms of support as needed. Such adaptive implementation strategies may be particularly suited to BITs and testing through factorial or sequential multiple assignment randomized trial designs [67]. In such trials, BIT Implementation researchers should clearly define the adoption, fidelity, or clinical outcomes that necessitate transitions.

### Downstream Effects on Health Service Utilization

One implicit assumption of those advocating the use of self-care BITs through stepped care models is that the use of such services represents an alternative pathway or entry point to care [1]. Although we support this position, we believe analyzing the impact of BIT use in addressing unmet need and subsequent or alternative care utilization is an important area of further research. For instance, there may be sunk or opportunity costs of time for consumers who initially engage in a BIT and have difficulty, subsequently dropping out [68]. Such a failure may engender an attitude of therapeutic nihilism on the part of the consumer or even provider, who may lose motivation and limit further engagement in behavioral treatment [69]. However, such potential costs are largely theoretical and deserve further research as initial evidence has shown that engagement in face-to-face care may increase for consumers who also receive a BIT [15,70].

## Conclusions

BITs are part of the current patient-centered transformation in health care delivery but initial implementation attempts have had varied outcomes, and more rigorous measurement of BIT implementation is needed to advance the understanding of factors related to successful implementation. Such implementation outcomes must be recharacterized to account for the unique aspects of BITs, as Proctor et al suggested in their original work [23]. Through this recharacterization, we have identified areas where the field of BIT implementation science can be advanced further. More work, interaction, and debate in these areas will allow for the exploration of the empirical boundaries primarily between BIT’s development- related outcomes such as usability and implementation- related outcomes such as acceptability, feasibility, and appropriateness. Further studies can differentiate the conceptual and practical differences in these constructs as well as quantify hypothesized associations between them. Future work should also more clearly and empirically define the outcomes of adoption and fidelity with respect to BITs. Finally, work aimed at developing, defining, and testing strategies for implementing different BITs in varying contexts is needed. Our recharacterization of implementation outcomes with respect to BITs intends to potentiate this work.

## Abbreviations

BIT: behavioral intervention technology
MOOI: massive open online intervention
TAM: technology acceptance model
